# Oncologists’ and family physicians’ views on value for money of cancer and congestive heart failure care

**DOI:** 10.1186/2045-4015-2-44

**Published:** 2013-11-18

**Authors:** Dan Greenberg, Ariel Hammerman, Shlomo Vinker, Adi Shani, Yuval Yermiahu, Peter J Neumann

**Affiliations:** 1 Department of Health Systems Management, Faculty of Health Sciences & Guilford Glazer School of Business and Management, Ben-Gurion University of the Negev, P.O.Box 653, Beer-Sheva 84105, Israel; 2 Center for the Evaluation of Value and Risk in Health, Tufts Medical Center, Boston, MA, USA; 3 Chief Physician’s Office, Clalit Health Services Headquarters, Tel-Aviv, Israel; 4 Oncology Institute, Sheba Medical Center, Tel-Hashomer, Israel

## Abstract

**Background:**

Previous studies suggest that cancer-related interventions are valued by policy makers more favorably than interventions for other medical conditions, but the views of practicing physicians have not yet been assessed in Israel. Attitudes and judgments of practicing physicians may assist decision-makers in their deliberations on coverage of new technologies. We conducted a national survey in Israel among oncologists and family physicians to explore their views on access to care, coverage decisions and treatment recommendations for cancer and congestive heart failure (CHF) patients.

**Methods:**

We administered a web-based survey to 300 family physicians and 156 oncologists. The questionnaire included 24 statements and physicians were asked to indicate their level of agreement with each statement on a 5-point Likert scale, ranging from *“strongly agree”* to *“strongly disagree”*. Where relevant, physicians were asked to express their views on interventions for cancer and CHF respectively.

**Results:**

Response rates were 39% for family physicians and 36% for oncologists. Participants expressed similar views on cancer and CHF care and no significant differences were found between the two medical specialties. More than 85% of physicians believe that inclusion of a treatment in the National List of Health Services (NLHS) strongly affects their patients’ access to care. Approximately 80% suggest that more use of comparative-effectiveness and cost-effectiveness analysis is needed in coverage decisions. The vast majority of respondents (75%) suggest that assessment of value-for-money should be made by an independent (academic) institution or the national committee responsible for recommending coverage decisions, Seventy percent believe that treatments not included in the NLHS should be included in supplementary health insurance programs and only a small minority of respondents (<30%) believe that cancer-related interventions should receive higher priority than non-cancer interventions in coverage decisions.

**Conclusions:**

Our findings suggest that both oncologists and family physicians value cancer and CHF interventions equally. We could not find evidence for a “cancer premium” as implied from previous surveys and analysis of coverage decisions in various countries.

## Background

Innovative interventions in medicine may improve patients’ survival and/or quality of life (QoL), but such improvements frequently come at a substantial cost. Among health interventions, the cost of cancer treatment has received increased attention in the last decade mainly due to the very high treatment costs associated with newly developed chemotherapies and biological drugs [1-4]. The debate over cancer drugs has focused not only on the costs of treatments, but also on their relatively modest benefits, as many new drugs, such as those for patients with metastatic disease produce relatively small gains in life-expectancy or QoL [1,2].

The access to, and affordability of, new expensive anti-cancer drugs is of concern to patients, decision-makers and the general public [5-12]. Since many cancer drugs are so expensive, the vast majority of patients cannot afford to pay for them themselves. Acknowledging the unique circumstances of end of life care, and the high cost of new cancer interventions which is beyond the means of most patients, several jurisdictions and policy makers have adopted special mechanisms for coverage and reimbursement decisions on cancer drugs. Reimbursement agencies, like the National Institute for Health and Care Excellence (NICE) in England, tend to use more flexible criteria to value cancer drugs, even when their cost-effectiveness ratio is higher than their implicit or explicit threshold that determines “good value for money” [5,12].

As of 1995, Israel has a National Health Insurance Systems that provides universal coverage for every citizen or permanent resident who are free to choose from among four competing non-profit health plans. The National Health Insurance Law stipulates a National List of Health Services (NLHS) which all residents are entitled to from their health plans. Every year as part of the annual budgeting process, the government determines the additional budget that will be available to fund new technologies. The budget allocated is far from being sufficient to keep up with the pressures of the growing health care market, which makes priority setting and rationing inevitable. The recommendations on which new technologies should be added to the NLHS are made by a Public National Advisory Committee (PNAC). The committee evaluates all proposed technologies considering clinical, economic, social and ethical aspects. Following the governmental approval of the PNAC prioritized list of new technologies, health plans are required by law to supply the entire demand for these technologies [13-18]. All cancer-related drugs listed in the NLHS are not subject to patient copayment, whereas a copayment is required for drugs for most other diseases. Individuals that wish to be treated with medications that are not in the NLHS formulary have to cover the costs, either through out-of-pocket payments or via private health insurance.

In general, drugs and other interventions that are not included in the NLHS may be included in supplementary insurance programs offered by health plans to their members, or by for-profit commercial health insurance programs. However, as of 2007, the health plans’ supplementary insurance programs are prohibited from offering coverage for cancer medications and interventions [19]. The reason for this prohibition was a concern that several drugs not included in the NLHS will be available only to those that purchased voluntary health insurance, thus creating two ‘classes’ of public healthcare.

Israel is considered an early adopter of many technologies, granting public funding prior to their coverage in other healthcare systems. Among disease types, the proportion of the budget allocated to fund new drugs for treating life-threatening oncology diseases is substantial and exceeds other therapeutic areas [20]. This might imply that decision-makers in Israel give a higher priority to interventions aimed at treating cancer patients rather than other life-threatening medical conditions. Although the coverage decision process in Israel does not explicitly use results from cost-effectiveness analysis [17,18], studies have suggested that in many cases, the recommended technologies for inclusion in the NLHS are those with favorable cost-effectiveness results [21]. Nevertheless, the maximum societal willingness to pay for a life year or a quality adjusted life-year (QALY) gained has not yet been determined by policy makers in Israel.

Advanced congestive heart failure (CHF) is similar to metastatic cancer, in that both are life-threatening medical condition. Although drug therapy for CHF may be substantially cheaper than cancer drugs, the cost per patient of several implantable devices such as left ventricular assist devices (LVAD) and cardiac resynchronization therapy (CRT) devices may exceed $US100,000 and their cost-effectiveness remains uncertain [22,23].

### Oncologists and family physicians attitudes towards the cost of cancer care

Practicing oncologists are frequently on the front line of the controversy over expensive cancer treatments, having to decide whether to offer their patients new and expensive treatments, sometimes not included in the NLHS. Very often, these decisions are complicated by the financial burden such treatments place on patients and their families as their costs might be beyond many people’s means [24,25]. For example, two drugs included in the 2013 update of the NLHS (Vandetanib for medullar thyroid cancer and Vismodegib for basal cell carcinoma) cost more than $US100,000 for an average patient treatment protocol. Although oncologists play a fundamental role in treatment decisions, little is known about Israeli oncologists’ attitudes towards cancer costs, their beliefs about whether costs influence their prescribing of non-reimbursed drugs, and their comfort and readiness to make such decisions. We also know very little about oncologists’ beliefs regarding the use of cost-effectiveness information or their views on policies regarding access and reimbursement. Only recently, several studies from the U.S. and Canada have examined American oncologists’ attitudes on those aspects [26-29]. Similar to oncologists, family physicians may also be involved in their patients’ decisions whether to opt for care of very costly treatments, sometimes with only marginal benefits. As they are exposed to a wide variety of medical conditions and perhaps because of other factors, family physicians may have different views on health interventions than those possessed by oncologists. Attitudes and judgments of practicing physicians may assist decision-makers in their deliberations on coverage of new technologies and whether treatments for cancer, for example, should be assessed using different criteria and receive a higher priority, as compared with other interventions. We therefore conducted a national survey in Israel among oncologists and family physicians to explore their views on access to care, coverage decisions and treatment recommendations for cancer and congestive heart failure.

## Methods

### Survey development

We developed a questionnaire to assess oncologists’ and family physicians’ views on various aspects of cancer and congestive heart failure treatment costs, cost-effectiveness (value for money), patients’ access to care as well as views on health policies relating to coverage and reimbursement decisions for these treatments. To allow comparison with findings from other healthcare systems [26-29], the questionnaire was partially based on a survey of oncologists in the United States and Canada. Several questions included in the original surveys were not relevant to the practice in Israel and were therefore omitted. We added questions specifically relating to the decision-making process regarding coverage of new technologies as well as inclusion of interventions not granted public funding in private health insurance programs (supplemental health insurance offered by health plans, or private insurance offered by commercial health insurance companies). Physicians were asked to indicate their level of agreement with each statement on a 5-point Likert scale, ranging from *“strongly agree”* to *“strongly disagree”*.

Where relevant, respondents were asked to express their views on both interventions for cancer and congestive heart failure. For example: all physicians were asked to comment on the following statements:

“Every patient should have access to effective cancer treatments regardless of their cost.”

“Every patient should have access to effective congestive heart failure treatments regardless of their cost.”

As oncologists treat only cancer patients they were not asked to express their views on questions relating directly to treatment of CHF patients (e.g., ”*whether the treatment for congestive heart failure is included in the National List of Health Services in Israel highly influences my patients’ access to this treatment*”).

The questionnaire administered to oncologists included 22 statements, and family physicians were asked to comment on 24 statements.

We also asked respondents what they thought a reasonable definition of “good value for money” or cost-effectiveness per life year gained is? (Response categories were $0-25,000, $25,001-50,000, $50,001-75,000, $75,001-100,000, $100,001-125,000, $125,001-150,000, $150,001-175,000, 175,001-200,000 and more than $200,000, and reflect the maximum willingness to pay or threshold per life-year). We also questioned them about who they think should determine whether an intervention provides good value for money. Possible answers included the Public National Advisory Committee (responsible for recommending which technologies should be included in the NLHS), an independent academic or research institution, the Ministry of Health or the Ministry of Finance, the health plans, private health insurance companies, or the patients themselves. Finally, we collected physicians’ demographic data and information on medical training and practice.

### Study population and survey administration

We sent the questionnaire to 156 board-certified oncologists with a valid email address and to a randomly selected sample of 300 board-certified family physicians received from the Israel Association of Family Physicians. The list of oncologists was compiled based on the directory of the Israeli Society of Clinical Oncology and Radiotherapy (ISCORT) and from the list of physicians practicing in general medical centers and in the Israeli four health plans.

The survey questionnaire was developed and distributed using the Qualtrics Online Survey Software (Qualtrics Labs, Inc. Provo, Utah, USA). Subsequent to pilot-testing for clarity and technical properties, participants received an e-mail invitation to complete the web-based questionnaire and a link to the questionnaire. To avoid ordering bias, each participant received and answered the above-mentioned questions in a random order. Differences in oncologists’ and family physicians’ views and training characteristics were assessed using chi-square tests. All analyses were performed using PASW Statistics 18. P values < 0.01 were considered statistically significant for all comparisons.

The study was approved by the ethics committee of the Faculty of Health Sciences of Ben-Gurion University of the Negev.

## Results

We received responses from 52 oncologists and 116 family physicians for overall response rates of 36% and 39% respectively. Ten out of the 156 oncologists did not practice medicine in Israel and were therefore excluded from our study population. Respondent demographics and medical training characteristics are presented in Table 1. Approximately 50% of respondents were male; oncologists were older, had substantially longer experience in medical practice and approximately 40% had a fellowship training in the United States. The main practice setting of the vast majority of oncologists (87%) was in public hospitals, while almost all primary care physicians practiced medicine in one of the four health plans.

**Table 1:** Physician’s characteristics

	**Oncologists (N = 52)**	**Family physicians (N = 116)**	**P value**
Age (Mean ± SD)	54.6 (10.8)	50.1 (9.2)	0.006
Female	50.0%	51.7%	
Male	50.0%	48.3%	NS
Years (Mean ± SD) of work experience in Medicine	28.6 (11.2)	23.1 (9.5)	0.001
Medical School			
Israel	48%	57%	NS
Western Europe and America	13%	16%	
Eastern Europe	27%	24%	
Other	12%	3%	
Fellowship in the United States	39%	6%	<0.001
Additional training			
Health administration/public health	2%	11%	
Masters/Ph.D. in basic sciences	8%	3%	
Other		7%	
Employment type			
Salaried		64%	
Self-employed		7%	
Salaried and self-employed		27%	
Other		3%	
Main practice setting			
Health plan (HMO)			
Other			
Main practice setting			
Public hospital	86%	2%	
Health plan (HMO)		91%	
Private hospital	6%	0%	
Other		7%	
Type of cancer			
Breast	58%		
GI	56%		
Lung	37%		
Genitourinary	29%		
Gynecologic	21%		
Head and neck	21%		
Sarcoma	21%		
Melanoma	14%		
Hematologic	4%		
Other	8%		

### Physicians’ views on coverage and reimbursement of new interventions for cancer and congestive heart failure

Oncologists’ and family physicians’ views on the coverage and reimbursement decisions of new technologies and the related health policies are summarized in Table 2. Overall, oncologists and family physicians expressed similar views on these topics.

**Table 2:** Views on coverage and reimbursement decisions

	**% stating strongly or somewhat agree**		**P value**
	**Oncologists**	**Family physicians**	
New *cancer drugs* should receive a higher priority compared with treatment for other diseases in the deliberations of the Public National Advisory Committee in Israel	27%	30%	NS
New treatments for *congestive heart failure* should receive a higher priority compared with treatment for other diseases in the deliberations of the Public National Advisory Committee in Israel	17%	22%	NS
Only effective *cancer* treatments that provide “good value for money” should be included in the National List of Health Services	48%	42%	NS
Only effective treatments for *congestive heart failure* that provide “good value for money” should be included in the National List of Health Services	42%	42%	NS
Using data on the cost-effectiveness of *cancer drugs* to support decisions whether to include these drugs in the National List of Health Services should be encouraged	73%	77%	NS
Using data on the cost-effectiveness of *congestive heart failure* drugs to support decisions whether to include these drugs in the National List of Health Services should be encouraged	77%	81%	NS
Only *cancer drugs* that provide substantial survival gains in comparison with the current treatment should be added to the National List of Health Services	62%	52%	NS
Only treatments for *congestive heart failure* that provide substantial survival gains in comparison with the current treatment should be added to the National List of Health Services	56%	52%	NS
Only *cancer drugs* that provide substantial improvements in patients’ quality of life in comparison with the current treatment (and no survival gains) should be added to the National List of Health Services	53%	50%	NS
Only treatments for *congestive heart failure* that provide substantial improvements in patients’ quality of life in comparison with the current treatment (and no survival gains) should be added to the National List of Health Services	50%	54%	NS
Over the next five years, the high cost of new *cancer drugs* will cause the Public National Advisory Committee to recommend the funding of only very few new treatments	62%	64%	NS
Who should determine whether a new intervention provided good value for money?			
Independent academic or research institution	44%	47%	
The Public National Advisory Committee	29%	30%	
Ministry of Health or Ministry of Finance	17%	14%	
Health plans	2%	3%	
The physician	6%	1%	
The patient	2%	5%	
Private health insurance companies	0%	0%	
Other		1%	
What do you think is a reasonable definition of “good value for money” or cost-effectiveness per life-year gained ($ per life-year)			
0–25,000	17%	35%	0.012
25,001-50,000	23%	29%	
50,001-75,000	15%	16%	
75,001-100,000	29%	10%	
>100,000	15%	10%	

Most respondents did not think that cancer and CHF interventions should receive higher priority in the PNAC’s deliberations. The majority of physicians believe that data on the cost-effectiveness of new interventions should be used to support whether these interventions should be included in the NLHS. Nevertheless, only less than half of respondents believe that only treatments that provide “good value for money”, i.e. are cost-effective, should be included in this list. The median range of cost-effectiveness threshold thought to reflect good value for money for cancer interventions ($US/life year gained) was lower for family physicians (25,000-$50,000), compared with oncologists (50,000-$75,000).

About half of respondents supported the statement that only interventions that provide substantial survival gains should be listed. A similar proportion of physicians believed that only interventions that offered improvement in patients’ quality of life (even with no survival gain) should be included in the NLHS. In terms of who they believe should determine whether an intervention provides good value for money, approximately half of Israeli physicians (regardless of type of medical specialty) favored having an academic or research institution, followed by the PNAC (30%), and the Ministries of Health and Finance (15%). Only very few suggested that these decisions should be made by health plans, the physicians or the patients themselves.

### Physicians’ attitudes regarding access to care and treatment recommendations

Physicians’ attitudes regarding access to cancer and CHF interventions and influences on recommended treatments are summarized in Table 3. Overall, oncologists and family physicians expressed similar views and concerns about access to care regardless of type of disease. Almost all physicians suggested that inclusion of an intervention in the NLHS in Israel highly influences their patients’ access to care. However, a lower proportion of oncologists and family physicians (54-77%) indicated that a drug listing in the NLHS would influence their treatment recommendations. Only very few physicians (10% of respondents) believe that patient co-payment for cancer drugs should be charged, even if the drug is included in the NLHS. More than two-thirds of physicians indicated that a treatment not included in the NLHS should be covered by either supplementary or by commercial health insurance programs.

**Table 3:** Attitudes regarding access to care and treatment recommendations

	**% stating strongly or somewhat agree**		**P value**
	**Oncologists**	**Family physicians**	
Whether a *cancer drug* is included in the National List of Health Services influences my decision regarding which cancer treatment to recommend to my patients	77%	54%	0.005
Whether an intervention for *congestive heart failure* is included in the National List of Health Services influences my decision regarding which treatment to recommend to my patients		65%	
Whether a *cancer drug* is included in the National List of Health Services highly influences my patients’ access to treatment	92%	84%	NS
Whether a treatment for *congestive heart failure* is included in the National List of Health Services highly influences my patients’ access to treatment		86%	
Every patient in Israel should have access to effective *cancer treatments* regardless of their cost	65%	55%	NS
Every patient in Israel should have access to effective treatments for *congestive heart failure* regardless of their cost	73%	59%	NS
Every effective *cancer drug* that is not included in the National List of Health Services should be included in the supplementary insurance offered by health plans	65%	76%	NS
Every effective treatment for *congestive heart failure* that is not included in the National List of Health Services should be included in the supplementary insurance offered by health plans	65%	78%	NS
Every effective *cancer drug* that is not included in the National List of Health Services should be included in commercial health insurance plans offered by private health insurance companies	75%	72%	NS
Co-payment on *cancer drugs* is needed even if the drugs are included in the National List of Health Services	10%	10%	NS
More research on comparative analysis of *cancer drugs* is needed	83%	85%	NS
Data on the cost-effectiveness of new *cancer drugs* may have an impact on which treatment protocol to recommend to my patients	52%	64%	NS
The notion that my patients will have to pay for a cancer drug “out of pocket” will influence my decision regarding which treatment protocol to recommend to my patients	79%	56%	NS
How often do you discuss the costs of *cancer drugs* with your patients?			
Always	6%	1%	0.007
Frequently	17%	14%	
Occasionally	50%	36%	
Rarely	27%	34%	
Never	0%	16%	
How often do you discuss the costs of treatments for *congestive heart failure* with your patients?			
Always		2%	
Frequently		16%	
Occasionally		30%	
Rarely		33%	
Never		19%	

A higher proportion of oncologists (77% of oncologists vs. 54% of family physicians; p = 0.005) suggested that the inclusion of a cancer drug in the NLHS will influence their treatment recommendations. Relatively few respondents (23% of oncologists, and 15% of family physicians) stated that they always or frequently discuss the costs of new cancer drugs with their patients, and only 18% of family physicians discuss with their patients the costs of CHF care.

## Discussion

Our study describes oncologists’ and family physicians’ attitudes toward the funding of and access to new interventions for cancer and congestive heart failure in the Israeli publicly funded health insurance system. This study adds to our previous analysis that examined how physicians value life-prolongation versus QOL-enhancing outcomes attributable to cancer and CHF interventions [30].

We were specifically interested in whether physicians believe that cancer interventions should be given “special care” as suggested in previous studies and analysis of coverage decisions in other jurisdictions. In addition to exploring the attitudes of oncologists, which has been studied in previous surveys in the U.S. and Canada, we also surveyed family physicians. As family physicians are involved in the treatment of a wide variety of patients and medical conditions, they may have more balanced views as compared with oncologists, who only care for cancer patients.

### Coverage and reimbursement decisions

Approximately half of the physicians favor an independent academic or research institution as the organization that should determine whether an intervention provides good value for money. The PNAC, currently responsible for recommending which technologies should be added to the NLHS, is less favored in completing this task. These views should be considered if cost-effectiveness analyses will become mandatory in making coverage decisions in Israel, as is already happening in several countries including the U.K, Australia, and Canada [5].

The majority of respondents in our survey felt that interventions not included in the NLHS in Israel should be covered under alternative complementary insurance plans. As mentioned earlier, although approximately 75% of the Israeli population is also covered by supplementary voluntary health insurance, the Ministry of Health banned the coverage of cancer drugs in these programs, although some plans already offered this benefit for their members for a relatively small premium. In return, the Ministry of Finance signed a three year agreement to allocate substantial funds for adding new technologies to the NLHS. The restrictions on supplementary health insurance plans were mainly due to equity concerns that created a situation where only patients with voluntary health insurance will have access to some of the important cancer medications. This decision has created major discussions and a debate among health plans, the Ministry of Health and patient advocate organizations. As this debate is still ongoing, the attitudes of the physicians in our study may serve as another argument for those objecting to this policy.

### Comparative effectiveness and cost-effectiveness

A large majority of physicians in our study indicated that more data on cost-effectiveness should be used to support coverage decisions on new cancer and CHF interventions considered for inclusion in the NLHS. A smaller, though still sizeable proportion of physicians (approximately 60%) suggested that data on the cost-effectiveness of new cancer drugs may influence their decisions on which treatment protocol to recommend to their patients. These results are somewhat interesting, as results from cost-effectiveness analyses are not currently used to inform coverage decisions in Israel. Moreover, practicing physicians are not regularly exposed to cost-effectiveness information and are not prepared to factor cost-effectiveness results into their treatment decisions. Our results are similar to attitudes revealed by Canadian and U.S oncologists (Table 4). Although cost-effectiveness analysis is used in Canada for making funding decisions on cancer drugs, U.S. policy makers are reluctant to use results from economic analyses, which may be viewed as an attempt to explicitly ration health care [5]. As suggested previously [27], one possible explanation for these unexpected findings is that physicians are beginning to acknowledge the unavoidable use of “value for money” considerations, given the relatively low benefits and very high costs associated with new cancer drugs.

**Table 4:** Oncologists’ views: comparisons across countries

**Statement**	**% stating strongly or somewhat agree**		
	**Israel**	**United States**	**Canada**
Only effective cancer treatments that provide “good value for money” should be included in the National List of Health Services*	44%	58%	75%
Every patient in Israel should have access to effective cancer treatments regardless of their cost	58%	67%	52%
Using data on the cost-effectiveness of cancer drugs to support decisions whether to include these drugs in the National List of Health Services should be encouraged*	76%	80%	69%
Co-payment on cancer drugs is needed even if the drugs are included in the National List of Health Services	10%	29%	41%
More research on comparative analysis of cancer drugs is needed	84%	79%	85%
Over the next five years, the high cost of new cancer drugs will cause the Public National Advisory Committee to recommend the funding of only very few new treatments*	63%	73%	NA
The notion that my patients will have to pay for a cancer drug “out of pocket” will influence my decision regarding which treatment protocol to recommend to my patients	63%	84%	80%
How often do you discuss the costs of cancer drugs with your patients?			
Always	2%	7%	7%
Frequently	15%	36%	41%
Occasionally	41%	37%	41%
Rarely	32%	17%	0%
Never	11%	3%	1%
What do you think is a reasonable definition of “good value for money” or cost-effectiveness per life-year gained ($ per life-year)			
0–50,000	57%	21%	12%
50,001-100,000	32%	49%	56%
>100,000	11%	30%	33%

Data for Israel is a summary of responses of all physicians (oncologists and family physicians). Data for the U.S. and Canada are summary of oncologists’ attitudes.

* Statements presented to physicians in Israel were slightly different than those presented to physicians in the U.S. and Canada, and were modified to reflect the practice and health insurance structure in Israel and the access to care granted through a National List of Health Services (NLHS) in Israel.

In terms of what cost-effectiveness thresholds were felt to represent “good value for money,” Israeli family physicians endorsed lower thresholds than oncologists. Moreover, both values were lower than cost-effectiveness thresholds endorsed by U.S. and Canadian oncologists; while in these countries approximately one third of physicians endorsed a value higher than $100,000 per life-year, only 11% of physicians in Israel suggested that this value is appropriate [30].

Research on comparative effectiveness has received in recent years an increasing priority in many Western countries; however, when cancer drugs are considered, informative head-to-head clinical trials which are most relevant to decision-makers and practicing physicians are seldom performed. Therefore, it is not surprising that, similar to attitudes expressed by oncologists in Canada and in the U.S., both oncologists and family physicians in Israel believe that more research on comparative effectiveness of cancer drugs is needed. As opposed to cost-effectiveness analyses that must be adapted individually to each healthcare system, data on comparative effectiveness can be shared and used across countries and jurisdictions.

### Access to care

A large majority of Israeli physicians indicated that out-of-pocket payment for cancer drugs will influence their decisions regarding which treatment protocol to recommend to their patients. In this regard, Israeli physicians share similar views with their U.S. and Canadian counterparts. Of note, the NLHS in Israel offers a generous coverage of cancer drugs and listed drugs are not subject to patient co-payment. Although almost every FDA approved cancer drug in the U.S. is covered by health insurance programs, copayments for these drugs frequently pose a very high financial burden on patients and their families. As some provinces in Canada do not cover some of the new and expensive cancer drugs, patients have been recently facing higher co-payment costs. Therefore, it seems that Israeli physicians were referring to those interventions not currently covered under the National Health Insurance.

It is also not surprising that Israeli physicians do not discuss the cost and the proportion of physicians that always or frequently discuss the costs of treatments with their patients is substantially lower than reported by their U.S. and Canadian colleagues, where co-payment on cancer drugs is prevalent (Table 4). Moreover, the proportion of oncologists reporting that they discuss the cost of treatment with their patients is substantially higher as compared with family physicians. This is not surprising as the final treatment recommendations for cancer patients, as well as their clinical and financial consequences, are made by oncologists and not by family physicians. Likewise, treatment recommendations for CHF patients are most likely made by cardiologists.

### Limitations

Our study has several limitations. First, the initial sample size was somewhat small compared with previous surveys of this kind conducted in the U.S. and Canada [26-29], partially because the number of board-certified oncologists is small. The modest response rate, despite our major efforts to contact and encourage physicians to respond to the survey, might mean that our results may be subject to response bias. As we did not have information on the characteristics of physicians in our sample frame, we were unable to compare characteristics of respondents and non-respondents and can not assure that those that answered our survey adequately represent physicians in term of demographic and practice characteristics. Indeed, a comparison of our physician sample and the characteristics of board-certified physician population published by the Israel Ministry of Health indicates that in the case both oncologists and family physicians the ratio of males to females is roughly 50/50, similar to the split of physicians in Israel. For family physicians, the age distribution of the sample is also similar to that of the population. However, in the case of oncologists, the sample appears to over-represent oncologists aged 65+, while under-representing those under 45. Finally, due to the relatively small sample size, we were unable to assess whether physician attitudes are influenced by their characteristics (e.g., age, gender, practice experience), as well as previous training in health policy and management and managerial experience.

## Conclusions

In general, oncologists and family physicians have similar attitudes on coverage and reimbursement decisions, as well as on treatment recommendations and access to innovative care. Although some minor differences in attitudes between these physician groups do exist, most of these differences can be considered as gradations of opinions rather than completely different outlooks. Moreover, physicians expressed similar views on cancer and CHF care. This implies that, at least in the opinion of practicing physicians, cancer interventions should not be given special care (i.e., apart from their relative cost and effectiveness) in coverage decisions, as is implicitly suggested by coverage decisions in Israel. A higher priority granted to cancer drugs in resource allocation decisions may lead to misallocation of resources. A recent analysis scrutinizing the NICE “end-of-life premium” in England, where special additional weight is given to health gains from life-extending end-of-life treatments (most frequently end-stage cancer patients), concluded that this policy may be inconsistent with any attempt to assess the value for money of public expenditure on medical technologies [12]. Whether cancer interventions should be granted a higher priority in the prioritization process or not should be also assessed among a sample of the adult population in Israel.

We believe that the views of practicing physicians in Israel should be given some weight in national coverage decisions, and specifically in the process of updating the NLHS. Currently, most professional medical societies, including The Israeli Society of Clinical Oncology and Radiotherapy (ISCORT) are approached and asked to prioritize the technologies proposed for inclusion in the NLHS in their relevant medical field. This ranking is given some weight in the deliberations of the PNAC and medical experts are consulted regarding specific technologies. We believe that family physicians that may have a broader perspective on various disease areas should also be approached and present their views to the committee, not only on specific technologies prescribed routinely by family physicians.

Physicians in Israel, as for their counterparts in the U.S. and Canada, believe that over the next years, due to the increasing costs of innovative cancer drugs, the coverage of only few cancer treatments will be possible. Coverage of new interventions will be also influenced by the recent austere conditions in many countries and the associated budget cuts. Under these conditions, choosing among those interventions with the highest efficacy and more specifically those that provide the best value for money becomes much more relevant. As Israel is considered an early adopter of new technologies, coverage decisions are currently made while supporting information on both the effectiveness and cost-effectiveness of new interventions is limited.

However, as suggested by physicians in Israel, Canada, and the U.S., more use of comparative effectiveness and cost-effectiveness studies would be beneficial. These and other considerations may be combined into a multi-criteria decision analysis approach as has been recently suggested [18].

## Competing interests

The authors of this manuscript declare that they have no competing interests regarding this study.

## Authors’ contributions

All authors have made substantial contribution to the design of this study and interpretation of its findings. YY was responsible for data collection. DG performed the statistical analysis and drafted the first version of the manuscript. The manuscript was revised based on comments received from all authors. The final version of this study has been approved by all authors.

## Authors’ information

Prof. Dan Greenberg (DG) is Associate Professor and Chairman of the Department of Health Systems Management at Ben-Gurion University of the Negev, Israel. He is also affiliated with the Center for the Evaluation of Value in Health at Tufts Medical Center, Boston, MA, U.S.A.

Dr. Ariel Hammerman (AH) is Director of the Pharmaceutical Assessment Department at the Chief Physician’s Office of Clalit Health Services Headquarters, Tel-Aviv, Israel.

Prof. Shlomo Vinker (SV) is Chairman of the Israel Association of Family Physicians, Associate Professor in Family Medicine, Former Head of the Department of Family Medicine, Sackler School of Medicine, Tel Aviv University, and a family Physician in Clalit Health Services, Israel.

Prof. Adi Shani (AS) is a senior physician at the GI Unit, Sheba Medical Center, Tel-Hashomer, Israel. He was previously head of the institute of oncology at Kaplan Medical Center Rehovot, Israel.

Yuval Yermiahu (YY) was a research coordinator at the Department of Health Systems Management, Ben-Gurion University of the Negev, Beer-Sheva, Israel.

Prof. Peter Neumann (PN) is Director of the Center for the Evaluation of Value and Risk in Health, Institute for Clinical Research and Health Policy Studies, Tufts Medical Center and Professor of Medicine at Tufts University School of Medicine, Boston, M.A, U.S.A.
